# Fluoride-induced testicular and ovarian toxicity: evidence from animal studies

**DOI:** 10.1186/s40659-025-00586-6

**Published:** 2025-01-25

**Authors:** Seyedeh Fahimeh Talebi, Mohammad Seify, Ramji Kumar Bhandari, Hamed Shoorei, Shahram Dabiri Oskuei

**Affiliations:** 1 Student Research Committee, Birjand University of Medical Sciences, Birjand, Iran; 2 Research and Clinical Center for Infertility, Shahid Sadoughi University of Medical Sciences, Yazd, Iran; 3 Division of Biological Sciences, University of Missouri, Columbia, MO 65211 USA; 4 Department of Anatomical Sciences, Faculty of Medicine, Tabriz University of Medical Sciences, Tabriz, Iran; 5 Clinical Research Development Unit of Tabriz Valiasr Hospital, Tabriz University of Medical Sciences, Tabriz, Iran

**Keywords:** Fluoride, Sodium fluoride, Testis, Ovary, Genes, Hormones

## Abstract

Fluoride (F), as a natural element found in a wide range of sources such as water and certain foods, has been proven to be beneficial in preventing dental caries, but concerns have been raised regarding its potential deleterious effects on overall health. Sodium fluoride (NaF), another form of F, has the ability to accumulate in reproductive organs and interfere with hormonal regulation and oxidative stress pathways, contributing to reproductive toxicity. While the exact mechanisms of F-induced reproductive toxicity are not fully understood, this review aims to elucidate the mechanisms involved in testicular and ovarian injury. In males, F exposure at different doses has been associated with reduced testis weight, reduced sperm quality in terms of count, motility, and viability, as well as abnormal sperm morphology and disruption of seminiferous tubules by altering hormone levels (especially testosterone), impairing spermatogenesis, and inducing oxidative stress and zinc deficiency. Similarly, administration of F can impact female reproductive health by affecting ovarian function, hormone levels, oocyte quality, and the regularity of the estrous cycle. However, the impact of F exposure on LH, FSH, and GnRH levels is controversial between males and females. In both males and females, F exerts its adverse effects by triggering apoptosis, autophagy, inflammation, mitochondrial dysfunction, reduction in ATP synthesis, and modulation of important genes involved in steroidogenesis. Furthermore, genetic susceptibility and individual variations in F metabolism may contribute to different responses to fluoride exposure.

## Introduction

**Fig. 1:**
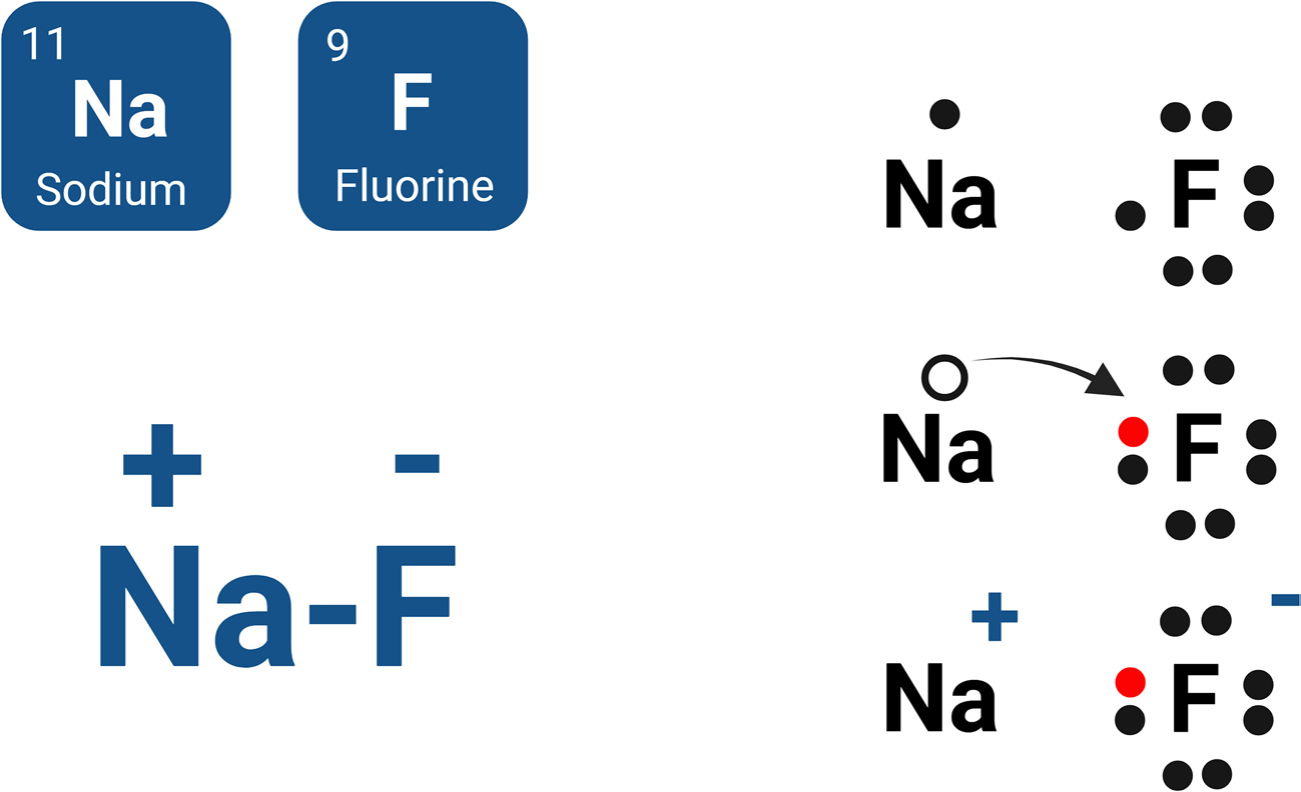
Sodium fluoride (NaF) formation

Since the 1940s, many countries have implemented the addition of fluoride (F) to drinking water as a preventive measure against dental caries [1]. This practice has generated significant debate regarding the potential risks and benefits of F, particularly due to the increasing incidence of fluoride-induced health issues [2]. Ranked among the twelve most hazardous elements, F possesses high electronegativity, making it the most chemically electronegative among all metallic elements. It exists primarily as a univalent gaseous halogen and can form ionized fluorides or inorganic salts, such as sodium fluoride (NaF) as a white or colorless compound (Fig. 1) [1, 3, 4].

In aqueous solutions, F ions act as enzyme inhibitors, with their effects influenced by factors such as concentration, exposure duration, and the specific cell type [4]. Ionized fluorides have been implicated in various tissue and organ damage due to their potential for accumulation in the body. The adverse effects of F exposure have been observed in over 200 million people worldwide, particularly in regions such as the United States, Saudi Arabia, India, and China, where conditions like fluorosis have become prevalent due to environmental factors, including coal-burning pollution [4, 5].

Exposure to F levels over 1.5 mg/L, primarily from contaminated drinking water, is associated with health issues such as skeletal and dental fluorosis, along with reproductive and neurological abnormalities in humans and animals [6]. In the body, the placental barrier, blood-testis barrier (BTB), and blood–brain barrier (BBB) are permeable to F [7–11]. Accumulations of F in various brain regions could disrupt biochemical processes related to energy metabolism and hormonal regulation within the hypothalamic-pituitary-gonadal (HPG) and hypothalamus-hypophysis-testis (HHT) axes.

The biochemical impact of F is significant; it inhibits enzymes involved in the Krebs cycle and glycolytic pathway by binding to amino acid groups at active sites, diminishing cellular respiration and ATP synthesis. This inhibition also affects Na + /K + -ATPases, resulting in ATP depletion and disrupted membrane potential [12]. NaF has been shown to induce the release of cytochrome-c (Cyt-c) into the cytosol, elevating cellular levels of inorganic phosphate (Pi), adenosine monophosphate (AMP), adenosine diphosphate (ADP), and guanosine diphosphate (GDP) [13]. The prolonged suppression of Cyt-c is particularly detrimental when glycolytic ATP synthesis cannot meet energy demands. Fluoride exposure also leads to significant DNA damage due to oxidative stress (OS), which is a primary cause of cellular toxicity [14–16]. This damage affects both single- and double-stranded DNA and can result in S-phase cell cycle arrest [17]. Additionally, F triggers endoplasmic reticulum (ER) stress, disrupting ER membrane integrity and calcium homeostasis, although the precise mechanism remains unclear [18]. The inhibition of Golgi stacking and protein transport is another consequence of F exposure, with aluminum fluoride (AlF4 −) activating G-proteins involved in signal transduction, impacting vesicle-mediated exocytosis and Golgi function [13, 19].

In the reproductive system, F disrupts spermatogenesis by traversing the BTB and altering G-protein signaling in Sertoli and Leydig cells [20]. This results in reduced production of key hormones, including estradiol (E2), progesterone (P4), thyroid hormones (T3 and T4), and testosterone (T), by impairing their receptors, *androgen receptor (AR) and* epidermal growth factor receptor (EGFR) [21–23]. NaF exposure can reduce pregnancy success rates, lead to a complete loss of sperm motility, and increase the abnormality of spermatozoa [23–25]. Studies have shown that F can disrupt thyroid hormone function, leading to decreased levels of sex hormones and inducing oxidative stress in the testes or ovary, ultimately resulting in abnormal spermatogenesis, folliculogenesis, and finally infertility [26–30]. NaF has been also linked to negative effects on female reproductive health, particularly by disrupting hormone levels through the HPG axis and decreasing gonadotropin-releasing hormone (GnRH) levels, which can lead to oxidative stress and ovarian cell apoptosis probably by accelerating the release of caspase-3/9 [31–35]. NaF exposure can also activate the nuclear factor kappa-light-chain-enhancer of activated B cells (NF-κB) pathway and increase nitric oxide (NO) production, leading to the expression of inflammatory cytokines such as tumor necrosis factor-alpha (TNF-α) and interleukin-1 beta (IL-1β) in testicular and ovarian tissues, resulting in sperm abnormality and ovarian dysfunction [35–37]. Moreover, in vitro studies have demonstrated that NaF can induce autophagy in Leydig and Sertoli cells as well as granulosa cells in a dose-dependent manner and cause mitochondrial dysfunction, leading to reduced mouse oocyte maturation and hormone production [38–40]. In addition, human studies have shown that F exposure can lead to a disruption of reproductive hormones (total T, sex hormone-binding globulin (SHBG), and E2) in men as well as women at different ages [41–46].

Despite these findings, this study aims to investigate how F contributes to ovarian and testicular injuries, exploring the underlying mechanisms involved.

## Fluoride exposer and oxidative stress induction

**Fig. 2:**
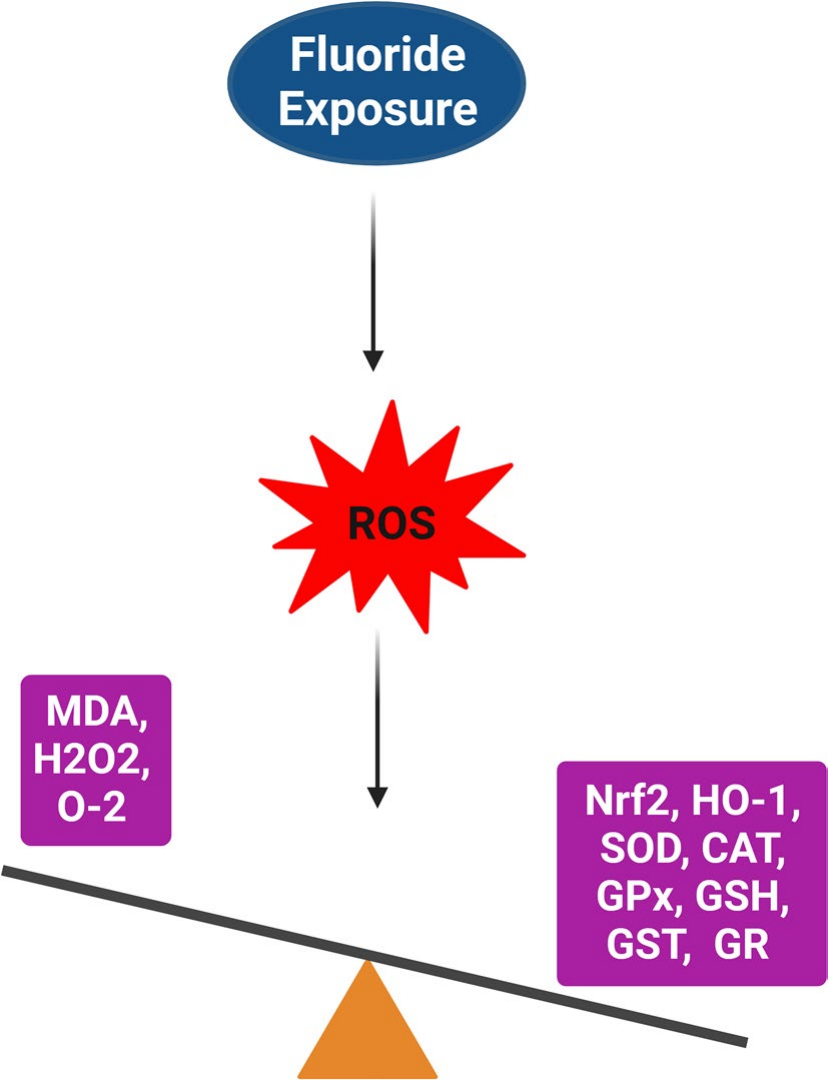
Fluoride exposure can disrupt the balance between ROS and antioxidant defense mechanisms within cells

Numerous studies on both humans and animals have highlighted the reproductive toxicity of F, primarily through the generation of free radicals and reactive oxygen species (ROS). In a study by Sun et al. (2018), exposure to NaF resulted in decreased expression of antioxidant enzymes in the epididymis of mice, indicating that F may impair the antioxidant defense mechanisms in reproductive tissues (Table 1) [47]. The body's response to ROS involves a robust defense mechanism comprised of enzymatic actions, including glutathione peroxidase (GPx), glutathione S-transferase (GST), superoxide dismutase (SOD), and catalase (CAT) [48]. However, F exposure has been shown to reduce the levels of these crucial antioxidants while increasing malondialdehyde (MDA), a marker of oxidative damage (Fig. 2) [35, 47, 49–55]. This disruption indicates an imbalance between ROS and the antioxidant defenses within cells.

**Table 1:** Fluoride exposure, oxidative stress markers, and genes involved in apoptosis, inflammation, and autophagy

Animal species	NaF doses, duration, and route of administration	Oxidative stress markers	Genes involved in apoptosis, inflammation, autophagy	Refs.
(Male): mice, rats, golden hamsters	NaF (1–100 mg/L or mg/kg, 0–300 ppm), Duration (daily, 3 to 26 weeks), Administration (oral gavage or drinking water)	Down: (SOD, CAT, GST, GSH, GPx, 8-OHdG) Up: (iNOS, NO, MDA, LPO)	Down: (Nrf-2, HO-1, SOD1, CAT, Bcl-2, SIRT-1, GPx-4, GPx-5) Up: (NF-κB, COX-2, IL-6, IL-17, IL-17A, IL-21, IL-23, TNF-α, IFN-γ, TGF-β, IL-1β, Bax, Bad, Caspase-3/7, Fas, GRP78, Cyt-c)	[36, 37, 47, 49–51, 63–65, 70–79]
(Female): mice, rats, zebrafish, drosophila melanogaster	NaF (10–100 mg/mL, 20–60 mg/L, and 200 ppm), Duration (daily, 20 to 60 days), Administration (oral gavage or drinking water)	Down: (GSH, SOD, CAT) Up: (MDA, H_2_O_2_)	Down: (SOD1, SOD2, GPx-1, Bcl-2) Up: (NF-κB, TNF-α, IL-6, Bax, p53, FasL, Caspase-3/8/9, Cyt-C)	[35, 54, 80, 81]

SOD, particularly its isoenzymes (SOD1, SOD2, and SOD3), plays a vital role in converting harmful hydrogen peroxide (H_2_O_2_) and superoxide anion (O − 2) into harmless molecules, O_2_ and H_2_O [48, 56, 57]. Inhibition of SOD by F can lead to the accumulation of superoxide, resulting in mitochondrial dysfunction and cell death [58]. Also, while chronic F treatment did not significantly alter SOD activity in some studies, NaF exposure has been associated with increased MDA levels and decreased glutathione levels (glutathione [GSH] and glutathione reductase [GSH-R]) in various animal models [59, 60]. The presence of MDA in sperm samples serves as an indicator of peroxidative damage, correlating with reduced motility and viability [61]. Therefore, this imbalance indicates insufficient levels of enzymatic and non-enzymatic antioxidants to effectively counteract free radicals induced by F exposure. Moreover, GSH is a critical antioxidant that interacts with oxidizing compounds. Its depletion is a key indicator of oxidative stress. The balance between GSH and its oxidized form (GSSG) is crucial for cellular health, and any disruptions can exacerbate oxidative stress [62]. The conversion of reduced GSH to oxidized GSSG involves the action of GSH-Px, which reduces lipid peroxides and H_2_O_2_. Conversely, the conversion of GSSG back to GSH requires GSSG reductase (GR) and utilizes nicotinamide adenine dinucleotide phosphate (NADPH) as an energy source [62]. Additionally, organic peroxides (ROOH) can be reduced by either GPx or another enzyme called GST. The inhibition of intracellular antioxidant status such as GSH suggests that F-induced oxidative stress is a potential mechanism underlying the toxic effects of F on reproductive organs [63]. In addition, catalase also plays a crucial role in breaking down hydrogen peroxide, but its activity can be inhibited by superoxide radicals [53]. The decline in CAT activity following NaF exposure suggests that F may inhibit both enzymatic and non-enzymatic antioxidants, further contributing to oxidative stress in reproductive organs.

Research by Thangapandiyan and Miltonprabu observed that F exposure downregulated the expression of key antioxidant defense genes, such as Nuclear Factor Erythroid 2-related Factor 2 (Nrf2) and heme oxygenase-1 (HO-1), while simultaneously overexpressing Kelch-like ECH-associated protein 1 (Keap-1), a repressor of Nrf2 [64]. This dysregulation may increase the oxidative burden on the testes, negatively affecting reproductive health. In hamsters exposed to NaF, researchers noted inhibited oxidative sensor markers like Silent Information Regulator Transcript-1 (SIRT-1) and Forkhead Box Transcription Factor O-1 (FOXO-1), both of which help protect cells from stress-induced inflammation and apoptosis [65]. Furthermore, NaF exposure activated inflammatory markers and downregulated antioxidant pathways, leading to increased oxidative stress and apoptosis in reproductive tissues. The transcription factor Nrf2, crucial for promoting antioxidant enzymes such as SOD, HO-1, and GSH, was also inhibited by F exposure, further exacerbating oxidative stress. NaF exposure also could inhibit cellular antioxidant markers such as Nrf2 and HO-1 and activated inflammatory markers, cyclooxygenase-2 (COX-2), and NF-κB, leading to apoptosis [65–67]. Additionally, Fluoride's impact on oocyte quality has been demonstrated through mechanisms involving SIRT-1 and SOD2, indicating that F may reduce oocyte quality via oxidative stress pathways [54]. In an in vitro study on chicken embryonic gonads, F treatment led to increased expression of free radical scavenging enzymes such as Nrf2 and SOD, affirming its role in inducing oxidative stress [53]. Moreover, NaF exposure can trigger ROS production from immune cells such as macrophages and neutrophils, negatively impacting sperm function [36]. The level of ROS generated correlates positively with F dosage, leading to the formation of defective sperm characterized by morphological abnormalities and activation of apoptotic pathways [68]. The lipid composition (polyunsaturated fatty acids) of sperm membranes makes them particularly susceptible to damage from ROS, and by disrupting the axoneme structure resulting in reduced motility and fertility [69]. Sperm DNA integrity is also compromised by oxidative stress, leading to higher frequencies of abnormal sperm and decreased sperm count. It has been shown that NaF exposure at high concentrations can result in severe sperm deformities, including broken heads and other malformations [70]. These deformities are linked to reduced levels of GPx-4 (glutathione peroxidase-4), an important antioxidant enzyme associated with maintaining sperm quality. Further research has demonstrated that F exposure increased markers of oxidative damage, such as 8-hydroxy-2'-deoxyguanosine (8-OHdG), while decreased levels of selenoprotein P (SePP), an extracellular antioxidant vital for sperm health [70].

In conclusion, F exposure significantly impacts reproductive health by inducing oxidative stress and disrupting the balance of antioxidant mechanisms. This leads to various sperm deformities and decreased oocyte quality, ultimately affecting fertility.

## Fluoride exposer, apoptosis, and mitochondrial dysfunction

**Fig. 3:**
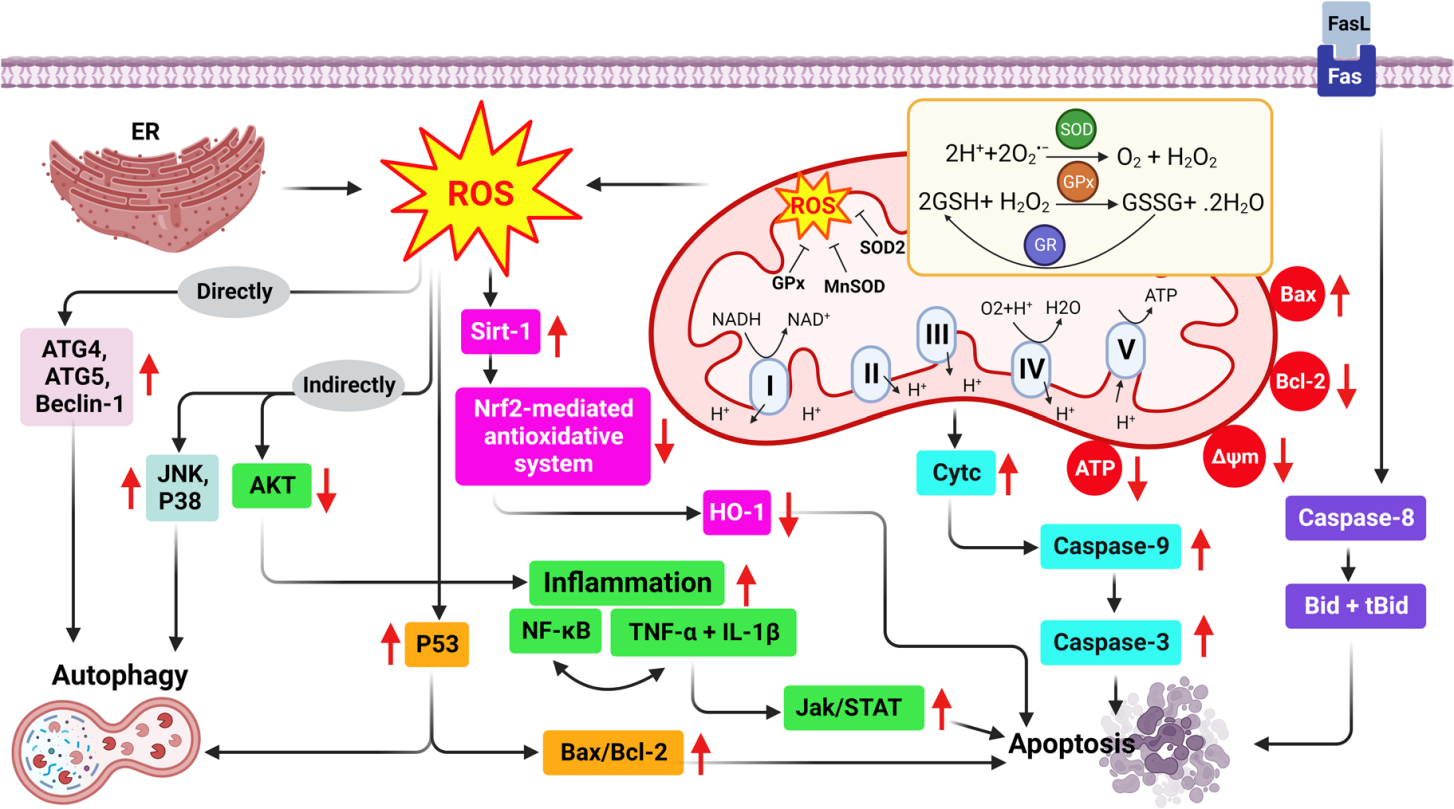
Fluoride exposure induces ROS, apoptosis, autophagy, and inflammation. Fluoride exposure can lead to excessive production of ROS and disrupt mitochondrial function, specifically mitochondrial respiration (ΔΨm), through the NADH oxidative respiratory chain, resulting in a decreased production of ATP. Furthermore, ROS can induce apoptosis, autophagy, and inflammation in ovarian and testicular cells through various pathways. Apoptosis can be triggered by the activation of cytochrome-c (Cyt c), caspase cascades, inflammatory cytokines such as *TNF*-*α* (by suppressing the AKT pathway), P53, and the suppression of the Nrf2/HO-1 pathway. Additionally, F-induced autophagy involves multiple pathways, including the direct pathway mediated by Beclin-1, the indirect pathway involving JNK and P38, and the involvement of P53

F exposure has been shown to adversely affect reproductive health by compromising mitochondrial function in both male and female reproductive systems (Fig. 3).

Transmission electron microscopy (TEM) has shown that F exposure causes mitochondrial vacuolation and ultrastructural damage [82]. In vivo and in vitro studies have documented that NaF exposure can activate both intrinsic and extrinsic apoptotic pathways in a dose- and time-dependent manner [52, 81, 83–87]. The interaction between F and BCL2 in the mitochondrial membrane facilitates the release of Cyt-c, leading to the activation of caspase cascades, testicular dysfunction, and ovarian atretic follicles [72, 87, 88]. Moreover, NaF can induce apoptosis through the TNF-R1 signaling pathway, involving the Fas-associated death domain (FADD), TNFR-associated death domain (TRADD), and Cyt-c [84, 89]. Furthermore, NaF has been shown to activate the apoptosis via the p53 pathway, the inositol-requiring enzyme 1/c-Jun N-terminal kinase (IRE1α/JNK) pathway while inhibiting Nrf2 [73, 90] (Table 1, Fig. 3).

In a study by Tan et al. [91], male C57BL-6 mice exposed to NaF (0–2.4 mM) for 3 months exhibited significant mitochondrial damage within the testes, accompanied by increased mRNA expression of ATP synthase subunit beta (ATP5B) [91]. ATP5B is essential for mitochondrial ATP synthesis, and its overexpression may lead to enhanced ROS production, resulting in mitochondrial dysfunction and testicular impairment. Similarly, Tang et al. [92] investigated the effects of NaF (200 mg/L) on silkworm testicular tissue, noting that prolonged exposure exacerbated tissue damage [92]. They reported increased expression of genes related to ATP synthase and heightened activity of mitochondrial complexes I, III, IV, and V [92]. The observed damage, including dilated endoplasmic reticulum, swollen mitochondria, and vacuole formation, was positively correlated with ROS accumulation, suggesting that the toxic effects of NaF are mediated through ROS activation and mitochondrial respiration via the NADH oxidative respiratory chain.

**Fig. 4:**
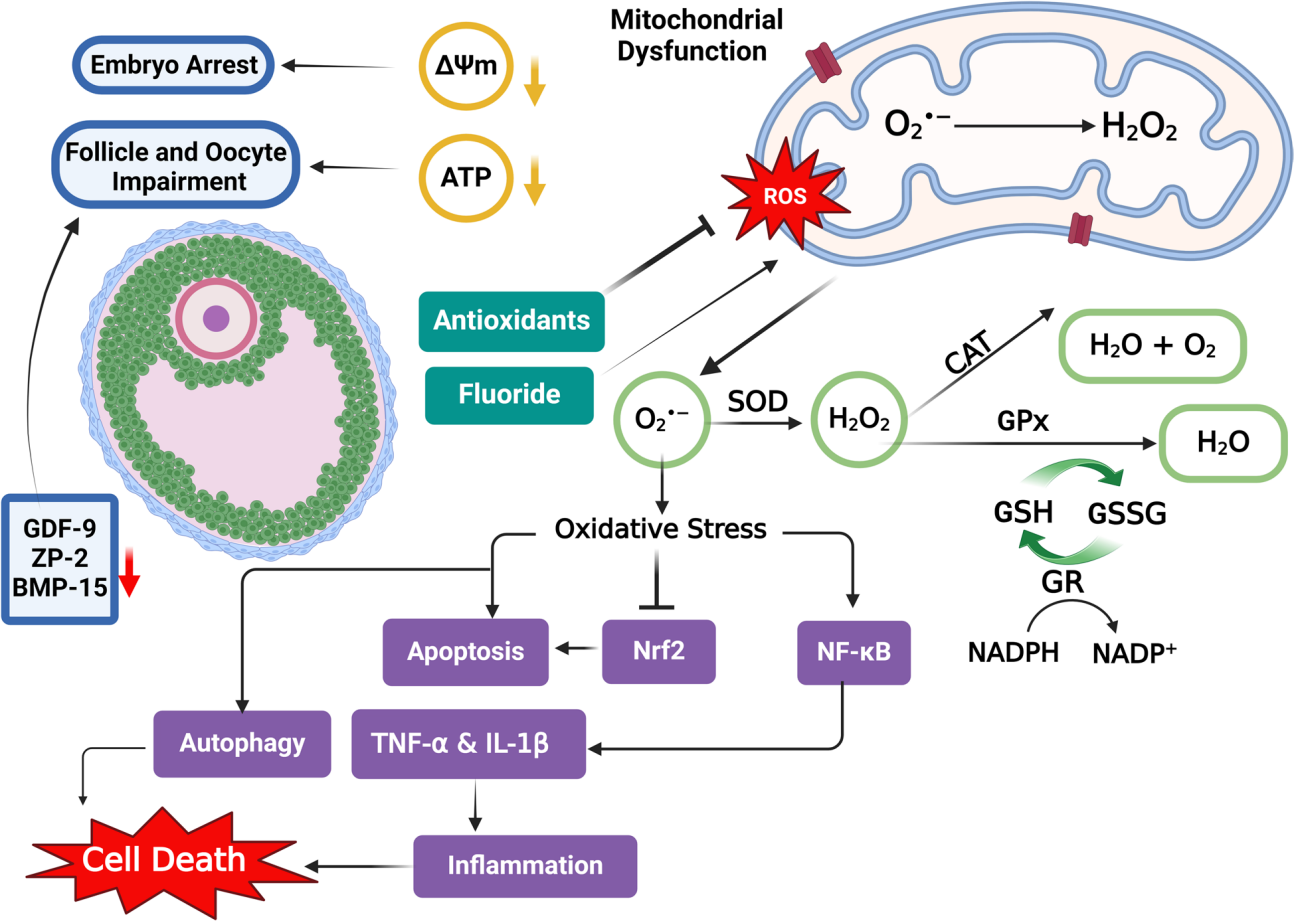
Fluoride exposure and ovarian dysfunction. Fluoride exposure induces oxidative stress, characterized by increased ROS, and mitochondrial dysfunction, evidenced by decreased ΔΨm and ATP levels. It also negatively impacts oocyte development, leading to reduced expression of key genes such as ZP-2, GDF-9, and BMP-15, and may result in embryo arrest. Additionally, it can activate critical pathways associated with apoptosis, inflammation, and autophagy, contributing to ovarian damage, as indicated by an increased number of atretic follicles. Conversely, antioxidant treatment has shown the potential to ameliorate these adverse effects of fluoride exposure

In females, mitochondrial health is critical during oocyte maturation. NaF exposure has been shown to decrease mitochondrial membrane potential (ΔΨm) in a dose-dependent manner, indicating impaired mitochondrial function [52]. This dysfunction can lead to developmental delays and embryo arrest (Fig. 4). Zhao et al. [82] further elucidated the impact of NaF on ovarian granulosa cells (GCs), revealing that NaF (25–100 mg/L) for 3 months downregulated the expression of ATP5j and ATP5h, which are vital for ATP synthesis through oxidative phosphorylation [82]. In contrast, the expression of NDUFV2 and SDHA, key components of mitochondrial respiratory chain complexes I and II, was upregulated [82]. This dysregulation in mitochondrial complex expression compromises mitochondrial function and impairs follicular and oocyte development [93–95].

Ultimately, NaF disrupts mitochondrial integrity by altering the activity of respiratory chain complexes, leading to increased ROS accumulation and reduced ATP production. These effects significantly compromise the overall health of the reproductive system.

## Fluoride exposure, inflammation, and autophagy

Inflammation can be triggered by various inducers, including toxic compounds and damaged cells. Numerous studies have demonstrated that NaF induces organ toxicity linked to inflammatory responses in animal models [37, 66]. Key inflammatory mediators, such as NF-κB and its downstream targets—including TNF-α, iNOS, IL-1β, and COX-2—play crucial roles in these responses [36, 96, 97]. NaF exposure stimulates the synthesis of pro-inflammatory factors like TNF-α, IL-1β, IL-17A, and NO, which can lead to abnormal autophagy and apoptosis in testicular cells, particularly germ cells (Fig. 3) [37, 98]. This regulation of pro-inflammatory cytokines is mediated through the PINK1/Parkin pathway [33, 77, 99]. In IL-17A knockout mice, NaF exposure significantly increased apoptosis in Leydig cells, resulting in reduced testosterone concentrations and compromised semen quality [33]. Thus, the testicular damage caused by NaF may be attributed to the overproduction of inflammatory cytokines (Table 1).

Autophagy, a vital cellular process for maintaining cell survival and recycling components, is also disrupted by NaF (Table 1). Studies indicate that NaF exposure impairs autophagic degradation in rat testes and ovarian granulosa cells [72, 87, 100]. In Sertoli cells exposed to various doses of NaF (0–0.5 mM for 24 h), increased mRNA expression of Beclin-1 and P62 indicates potential autophagy activation, while LC3 and Atg5 levels were decreased [38]. NaF exposure also by altering the expression of autophagy-related genes (LC3, Beclin-1, and Atg5) can be attributed to PI3K/AKT/mTOR pathway inhibition, resulting in cell death [39]. Moreover, exposure to NaF in human ovarian granulosa cells (KGN cells) induced autophagy in a dose-dependent manner, as evidenced by the increased expression of Atg5, Atg7, ULK, and P62 [87].

Therefore, fluoride exposure though inducing inflammation and autophagy can lead to ovarian and testicular dysfunction.

## Fluoride exposure and human studies

### Male studies

The number of studies shows the impacts of F exposure on human fertility in both sexes is limited. Two studies from India reported that F content in water from 1 to 14.5 mg/L can lead to a remarkable reduction of serum T levels [101, 102]. A study in Rajasthan found poorer sperm quality in populations with F levels (n = 75) exceeding 2 mg/L compared to non-endemic groups [103]. In Sri Lanka, Gulegoda et al. [61] reported higher serum F concentrations in male patients (n = 30) and decreased sperm count, motility, and viability in fluorosis-endemic areas [61]. He et al. [104] found that men with idiopathic infertility (n = 215) had higher sperm F levels (0.78 µg/L) than controls, suggesting a potential link to infertility (95% confidence intervals for semen F levels associated with oxidative stress) [104]. Chinoy and Narayana [105] showed that high F levels (250 mM) altered glutathione levels and reduced sperm motility [105]. Additionally, F exposure may interact with estrogen receptor-alpha (ESRα) gene polymorphisms to affect androgen-binding protein and SHBG levels in male Chinese farmers (n = 348, urinary F levels impact on serum SHBG levels [β =  − 0.060, 95% CI  − 0.101 to − 0.018]) [106]. SHBG aids the entry of sex hormones into cells, while androgen-binding protein transports and protects androgens, crucial for spermatogenesis [107]. Therefore, the variation in the impacts of F on androgen-binding protein levels is influenced by polymorphisms in the ESRα gene. This relationship indicates that F levels may have diagnostic and prognostic value in idiopathic male infertility.

### Female studies

Tuomivaara et al. [108] showed that in women residing in Northern California, maternal fluoride exposure, associated with oxidative stress pathways, resulted in significant alterations to the cord blood proteome during the second trimester [108]. There is a moderately strong positive correlation (r = 0.736) between the F concentrations in cord blood and maternal blood. In this regard, the average F concentration in cord blood is approximately 60% of that found in maternal blood. Malin et al. [109] investigated the association between F exposure and menarche age in the U.S. from 2013 to 2016, finding that higher water F levels correlated with menarche occurring 3.3 months earlier (β =  − 0.28, 95%CI − 0.54, − 0.02) [109]. Elevated plasma F levels were linked to a 5-month earlier menarche onset in non-Hispanic Black adolescents (β =  − 0.42, 95%CI − 0.62, − 0.22). Mechanistically, a study in rabbits demonstrated that F can disrupt adrenal gland function, leading to alterations in hormone regulation and production [110]. Additionally, F accumulation in the pineal gland may reduce melatonin production, which in turn can affect the regulation of reproductive cycles [111]. One investigation reported that prepubescent gerbils on a high-F diet exhibited lower melatonin levels and reached sexual maturation more rapidly than those on a low-F diet [112].

Liu et al. [45] found that childhood F exposure could significantly affect pubertal development in Mexican boys aged 10–17 (genital development [OR = 0.71, 95% CI 0.53–0.95]), not girls [45]. An earlier age at menarche was observed in girls from New York in relation to F exposure, according to one ecological study, whereas a different ecological investigation of Hungarian girls found no associations [113, 114]. Additionally, a correlation was noted between plasma and water F levels and sex steroid hormones in U.S. adolescents, indicating an age- and gender-specific inverse relationship with these hormones [46].

## Fluoride exposure and experimental studies

### Testicular histology

Numerous studies indicate that NaF exposure in a dose-dependent manner can significantly impact the structure and function of testicular tubules and accessory reproductive organs weight [50, 65, 73–75, 78, 115–118] (Table 2), although some studies report no effect on organ weights with short treatment durations (1.54, 50, and 100 ppm, for 1 month) [24]. Chronic NaF administration has been associated with severe testicular damage, including the loss of germ cells at various stages of spermatogenesis, leading to necrosis of testicular tubules and vascular hemorrhage [36, 51, 70, 119]. For instance, research by Chaithra et al. [50] demonstrated that male rats treated with NaF (1–10 mg/kg for 52 days) experienced a significant reduction in midpachytene and preleptotene spermatocytes and an increase in polyhedral Leydig cells [50].

**Table 2:** Effects of NaF Exposure on Testicular, Ovarian, and Oocyte Structure

Male animal species	Doses, duration, and route of administration	Testicular structure and germ cells	Refs.
(WT) C57BL/6 J Mice, Kunming Mice, Albino Mice, SD Rats, Albino Wistar Rats	NaF (0–100 mg/kg or mg/L, 0–600 ppm), daily, for 3–12.8 weeks, via drinking water and oral gavage	Decreased Weight (Body, Testis and Epididymis)**,** Decreased Number of Spermatogenic Cells, Absence of Spermatozoa, Edema, Mitochondria Swelling and Vacuoles, Germ Cell Apoptosis, Sever Testicular Amyloidosis, Disarranged Seminiferous Tubules, Hypocellular Interstitium, Necrosis, Vascular Hemorrhage, Seminiferous Tubule Atrophy, Absence of Elongated Spermatids, Unchanged Round Spermatid Counts	[33, 36, 50, 51, 64, 65, 70, 72–75, 78, 115–118, 141, 142]

Female animal species	Doses, duration, and route of administration	Ovarian and oocyte structure	Refs.
C57BL/6 Mice, Kunming Mice, SD Rats, Albino Rats, Wistar Rats	NaF (0–200 mg/kg or mg/L, 0–6 ppm), daily, for 1.1–12.8 weeks, via drinking water, oral gavage, and intraperitoneally	Decreased ovarian weight, Decreased Density of Granulosa Cells, Decreased Number of Mature Oocytes, Nuclear Pyknosis, Cloudy Zona Pellucida, Shrinkage of the Nucleus, Dilated Nuclear Membrane, Swollen Mitochondria and Endoplasmic Reticulum, Mitochondrial Crista Dissolution and Fracture, Abnormal Actin Cap Formation in Oocyte, Decreased Number of Super-ovulated Oocytes, Decreased Diameter of Oocytes, Decreased Blastocyst and Fertilization Rate, Decreased Numbers of Primordial, Primary, and Secondary Follicles, Increased Number of Atresia Follicles	[35, 54, 79, 82, 83, 130, 138, 139]

Mechanistically, NaF disrupts critical enzyme activities, including lactate dehydrogenase (LDH), acid phosphatase (ACP), and alkaline phosphatase (ALP), which are vital for spermatogenesis [65, 74, 120, 121]. Reduced LDH impairs lactate production necessary for germ cell health by changing the activity of specific glucose transporters, particularly GLUT-1 and GLUT-3, which are expressed in Sertoli cells, while decreased ACP and ALP levels may result from cellular necrosis, affecting membrane transport and cellular growth [74, 121–123]. Additionally, lower intratesticular T levels may trigger apoptosis in germ cells, further contributing to testicular degeneration and altered organ measurements following NaF exposure [124].

### Blood-testis barrier

NaF has been implicated in compromising the integrity of the BTB, potentially leading to testicular dysfunction [1, 65, 125]. The BTB is essential for creating a suitable microenvironment for spermatogenesis by segregating seminiferous tubules from systemic circulation [126]. Kumar et al. [65] demonstrated that F exposure induced structural changes in hamster seminiferous tubules, correlating with reduced expression of connexin-43, a vital gap junction protein [65]. Connexin-43 is crucial for maintaining BTB integrity and facilitates various cellular processes, including the maturation, differentiation, and proliferation of germ and somatic cells [127]. Consequently, the downregulation of genes essential for BTB integrity may adversely affect seminiferous tubule structure.

### Ovarian histology

Numerous studies indicate that NaF administration in a dose-dependent manner reduces ovarian, uterine, and vaginal weight in Wistar rats [35, 128–130] (Table 2). NaF also adversely impacts ovarian morphology, increasing apoptosis in Drosophila Melanogaster [80]. Observations in treated animals include extensive necrosis in the corona radiata and follicular antrum, atretic follicles (Fig. 4), dilated blood vessels, and the absence of zona pellucida [54, 83, 131].

The ovarian dysfunction resulting from long-term NaF exposure is likely linked to apoptosis induced by disrupting extracellular regulated protein kinase (ERK) and c-Jun N-terminal kinase (JNK) signaling pathways [132]. Additionally, NaF inhibits follicle formation and maturation by affecting the transcription of TIMP-4, MMP-9, and MMP-26 [133–135]. Matrix metalloproteinases (MMPs) are crucial for extracellular matrix remodeling, while tissue inhibitor of metalloproteinase (TIMP) regulates angiogenesis and trophoblast invasion during embryo implantation [136, 137]. In summary, NaF exposure leads to ovarian damage through multiple signaling pathways, compromising reproductive health.

### Oocyte function

In female mice, administration of NaF at concentrations of 50 to 200 mg/L for five weeks adversely affects both ovarian health and the number and fertilization potential of mature oocytes. This is primarily due to the downregulation of key genes essential for normal oocyte development, including Zona Pellucida Glycoprotein-2 (ZP-2), Growth Differentiation Factor-9 (GDF-9), Bone Morphogenetic Protein-15 (BMP-15), and Azoospermia Like (Dazl) [138]. The disruption of these genes hinders oocyte maturation, leading to reduced fertility (Fig. 4). NaF exposure also results in DNA damage in porcine oocytes, as indicated by increased levels of the biomarker γH2A.X [26]. This damage can impair meiotic competence, causing structural defects in spindle/chromosome formation, which may lead to aneuploidy and meiotic arrest [52, 54, 139]. Such abnormalities disrupt the asymmetric division critical for meiosis, affecting future embryonic development [140]. Additionally, treatment with NaF (2–10 mM) in cumulus-oocyte complexes induced the increase of DNA methylation of Neurontin (NNAT), associated with oocyte aging [139]. This epigenetic alteration reduces NNAT expression and impairs glucose transport in oocytes, further compromising oocyte quality and, consequently, embryonic development.

### Sperm function

**Fig. 5:**
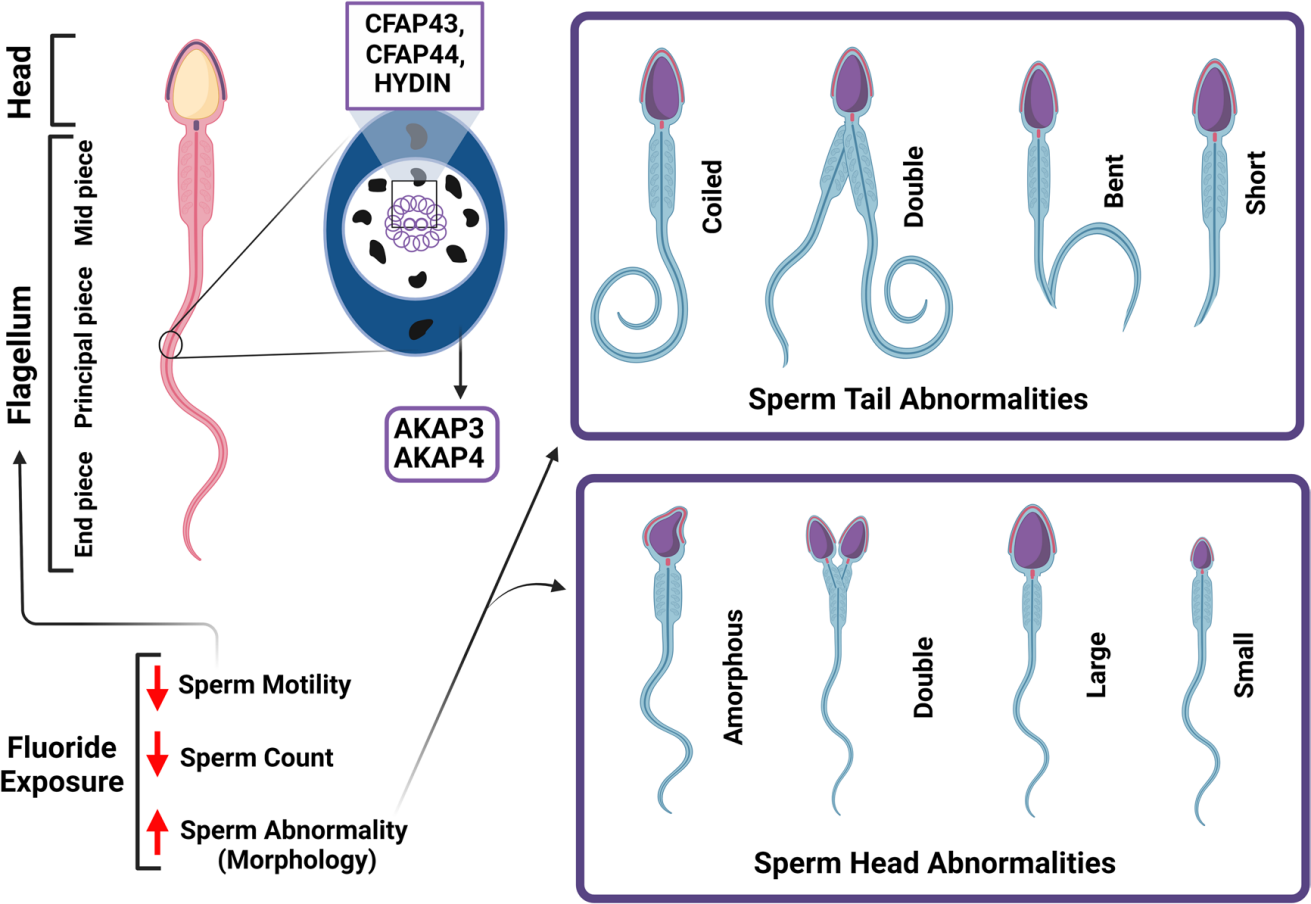
Fluoride exposure changes the viability, count, motility, and morphology of sperm. Fluoride exposure has been found to downregulate the expression of crucial genes in the testes, such as kinase anchoring protein 3 (AKAP3), AKAP4, cilia and flagella-associated protein 44 (CFAP44), and HYDIN protein [69]. These genes play essential roles in regulating sperm movement and flagellar function. For instance, in male mice lacking CFAP44, sperm motility is significantly reduced, leading to infertility [165]. Disruption of their expression through changing the structure of flagellar axons of spermatozoa can impair flagella movement and result in reduced sperm motility

Male sterility is intricately linked to sperm quality, and any alterations in this quality can significantly impair male fertility. NaF exposure has been shown to exert a dose-dependent detrimental effect on sperm quality, resulting in decreased sperm count, density, viability, motility, and increased rates of sperm deformity [36, 50, 63, 74, 76, 78, 117, 118, 143–147] (Table 3 and Fig. 5). These effects are partly attributed to NaF's ability to lower zinc levels in the testes, subsequently reducing angiotensin-converting enzyme activity, which ultimately disrupts normal sperm production [148, 149].

**Table 3:** Fluoride exposure and sperm parameters

Animal species	Doses, duration, and route of administration	Sperm parameters	Refs.
Guinea pigs, kunming mice, BALB/c mice, CD1 mice, Wistar rats	NaF (0–150 mg/kg, 0–600 ppm), daily, for 3–25 weeks, via drinking water and oral gavage	Up: (Slow Progressive Motility, Non-progressive Motility, Abnormal Morphology (Tail, Head, Neck defect), Sperm Deformity, Malformed Sperm) Down: (Count, Concentration, Viability, Progressive Motility, Normal Morphology, Acrosome Integrity, Plasma Membrane Integrity)	[36, 37, 50, 51, 63, 65, 71, 74–78, 117, 118, 145, 152, 166, 167]
Wistar rats	NaF, 4.5, 9.0 ppm, daily, GD (gestational day) to 90 PND (postnatal day), drinking water	Down: Count, Viable Sperm (all doses), Motile Sperm, Progressive (9.0 ppm)	[143]

Sperm morphology is a critical parameter that influences fertilization success. The toxic effects of NaF lead to an increased prevalence of abnormal spermatozoa, characterized by various head and tail defects [50, 63, 150] (Fig. 5). These abnormalities are linked to lipid peroxidation, mutagenicity, and cell membrane toxicity. However, some studies indicate that NaF does not induce significant changes in sperm morphology or production in certain animal models, suggesting variability in effects across different species or experimental conditions [24, 151].

Increased NaF intake has been associated with structural changes in spermatozoa that impair functionality [142, 152]. These changes include damage to cytomembranes and cytoskeletons, alterations in the ultrastructure of sperm flagella, and impaired mitochondria [69, 99, 153].

Proposed mechanisms by which NaF impacts sperm quality primarily affect motility rather than count include the downregulation of essential genes involved in regulating sperm movement and flagellar function, alterations to the epididymal environment critical for sperm maintenance and maturation, and interference with specific proteins like cysteine-rich secretory proteins 1 and 2 (CRISP1 and CRISP2), which are involved in sperm motility through activating calcium signaling pathways [47, 69, 154, 155]. Additionally, NaF disrupts energy metabolism by decreasing intracellular ATP production and binding to cofactors such as magnesium, calcium, zinc, and selenium, inhibiting glycolysis and respiration [63, 155–159].

NaF also disrupts spermatogenesis, particularly during the haploid stage, through the regulation of DEAD-Box Helicase 25 (DDX25), which is essential for the post-transcriptional regulation of spermatogenesis [142, 160]. Altered expression of DDX25 can lead to arrested progression of round spermatids, negatively impacting sperm quality [161]. Furthermore, NaF treatment downregulates the cAMP response element modulator (CREM) and its activator (ACT), both crucial for the transcription of post-meiotic genes necessary for normal spermatogenesis at the round stage of spermatid maturation [117, 162, 163]. Additionally, NaF exposure disrupts acrosome biogenesis, as demonstrated by Jiang et al. [164], who found alterations in the ultrastructure of the acrosome associated with downregulation of proteins like Zona Pellucida Binding Protein 1 (ZPBP1) and Sperm Acrosome Associated 1 (SPACA1) [164]. These proteins are vital for sperm-oocyte binding during fertilization.

In summary, F exposure significantly impairs sperm function, adversely affecting parameters such as motility, morphology, and overall quality. The mechanisms underlying these effects include disruptions in essential signaling pathways, oxidative stress, and alterations in sperm development processes.

## Fluoride and testicular and ovarian steroidogenesis

### Male studies

The proper functioning of the male reproductive system is closely linked to spermatogenesis and the regulation of steroid hormones, particularly testosterone (T). Follicle-stimulating hormone (FSH) and luteinizing hormone (LH) play crucial roles in this process by acting through the HPG axis. FSH stimulates Sertoli cells, while LH acts on Leydig cells to support spermatogenesis and maintain overall reproductive health [139].

**Fig. 6:**
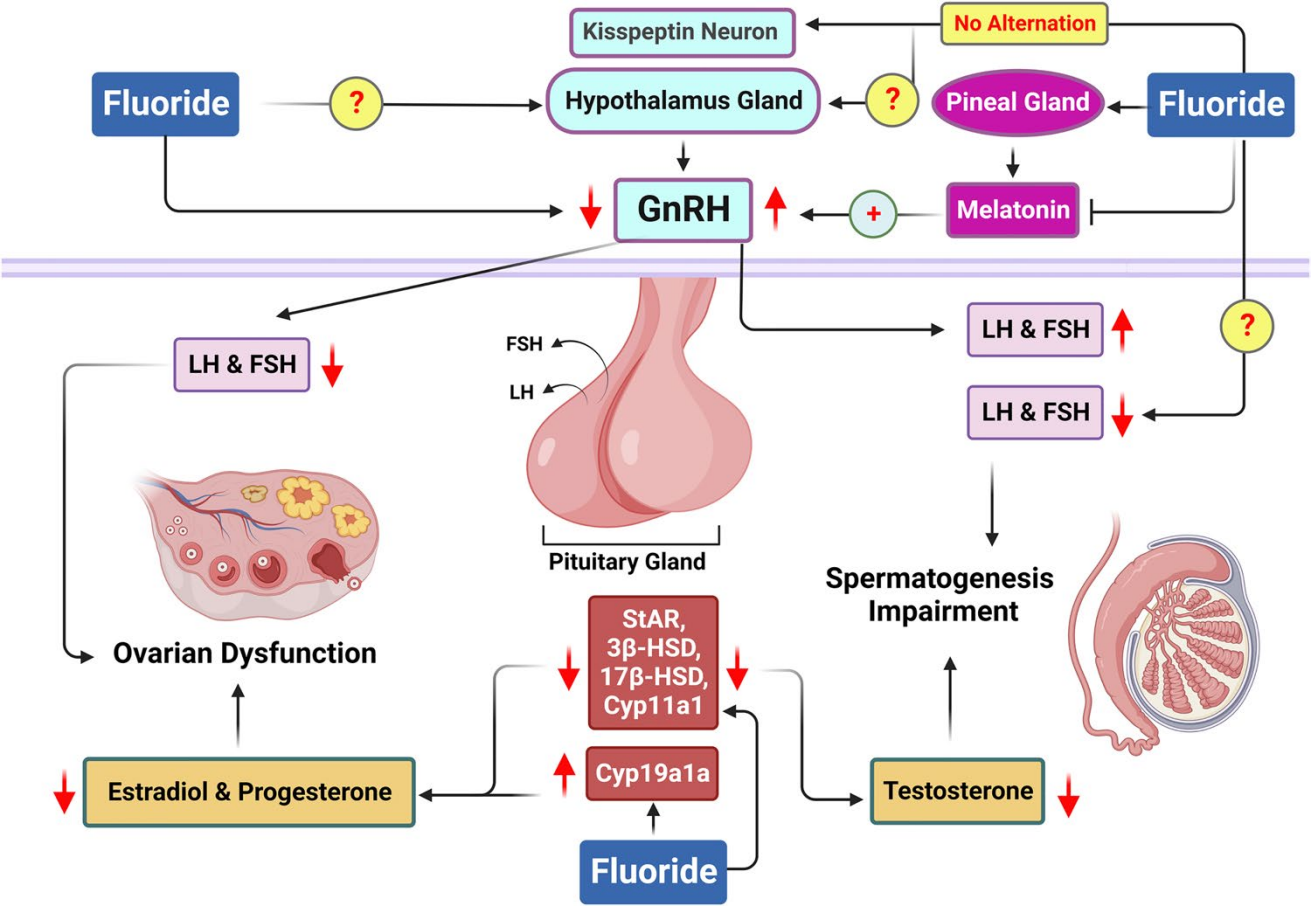
Fluoride exposure and steroidogenesis. F exposure disrupts the normal functioning of the HPG axis, although the precise mechanisms remain unclear. In males, F accumulation in the pineal gland reduces melatonin secretion, resulting in increased GnRH production and elevated serum levels of LH and FSH. However, the exact way F influences LH and FSH secretion, potentially by decreasing GnRH, is not yet fully understood. In females, F suppresses GnRH activation, leading to reduced LH and FSH production. Additionally, F interferes with key genes involved in steroidogenesis, such as StAR and 3β-HSD, diminishing the production of T, P4, and E2, which contributes to spermatogenesis impairment and ovarian dysfunction

NaF exposure disrupts the HPG axis, potentially lowering serum levels of T, E2, FSH, and LH [36, 159, 168]. While some studies report elevated FSH and LH levels due to F accumulation in the pineal gland—which inhibits melatonin release—melatonin typically exerts an anti-gonadotropic effect [9, 42, 169–172]. Thus, its suppression by F may lead to increased gonadotropin levels. Moreover, the regulation of GnRH appears less affected by F exposure than other hormones. The mRNA expression of kisspeptin-1 (KiSS-1) and its receptor, GPR54, which are critical for GnRH neuron function, remains stable after prolonged F treatment in animal models [173] (Fig. 6). Importantly, F exposure can induce mitochondrial damage, which may compromise T production since T biosynthesis occurs in the mitochondria [173]. This suggests that reduced T levels in male mice are primarily a result of F-induced mitochondrial dysfunction rather than direct interference with GnRH regulation.

F exposure also alters the expression of PIWI-interacting RNAs (piRNAs) and the JAK-STAT pathway, activating lysosomal signaling pathways in Leydig cells that are involved in T secretion [166, 174, 175]. Studies have documented decreased mRNA expression of key steroidogenic enzymes, including steroidogenic acute regulatory protein (StAR), cytochrome P450 cholesterol side-chain lyase (P450scc), 17β-hydroxysteroid dehydrogenase (17β-HSD), and 3β-hydroxysteroid dehydrogenase (3β-HSD) [49, 50, 70, 145, 153, 176, 177] (Table 4 and Fig. 6). This downregulation occurs in a dose-dependent manner and impairs spermatogenesis, attributed to altered expression of critical transcription factors such as Steroidogenic Factor-1 (SF-1) and Nuclear Receptor Subfamily 0 Group B Member 1 (Dax-1) [178]. Similar findings have been observed in zebrafish and pigs, where exposure to NaF resulted in decreased serum T levels and downregulated expression of StAR and P450scc, negatively impacting the synthesis of androstenedione and pregnenolone [70, 71, 179]. Excessive F intake has also been linked to reduced activity of enzymes involved in steroid hormone synthesis, specifically AKR1C3 and Cyp11a1, leading to inhibited T secretion [33, 180, 181].

**Table 4:** Fluoride exposure and hormones and genes involved in steroidogenesis

Animal species	NaF doses, duration, and route of administration	Hormones	Genes involved in steroidogenesis	Refs.
(Male): guinea pigs, rats, mice, and golden hamsters	NaF (1–150 mg/L or mg/kg, 0–300 ppm), duration (daily, 3 to 26 weeks), administration (oral gavage, drinking water, I.P)	Down: (T, LH, FSH, P4) Up: (LH, FSH), (The effects of NaF on LH and FSH are controversial)	Down: (StAR, P450scc, P450c17, 3β-HSDH, 17β-HSD) Up: (ER-α, ER-β, FSH-β) Did not change: (StAR, KiSS-1, GnRH, GPR54)	[36, 49–51, 64, 65, 70, 71, 73, 75, 78, 141, 143, 145, 166, 167, 170, 173, 188]
(Female): rats, zebrafish (Danio rerio)	NaF (5–200 mg/L or mg/kg), duration (daily, 4 to 12 weeks), administration (oral gavage or drinking water)	Down: (E2, P4, FSH, LH) Up: (E2)	Down: (LHR, 17β-HSDs, 3β-HSDs, AR) Up: (ERα, PgR, LHR, Cyp19a1a)	[23, 32, 131, 179, 184, 185, 187]

Disruptions in the normal functioning of androgen receptors and G proteins further compound the issue, as these are critical for effective T synthesis in Leydig cells [20]. Experimental evidence suggests that F negatively impacts G-protein function and suppresses AR and EGFR transcription, contributing to a decrease in T levels [65, 161, 182].

Therefore, NaF exposure can alter the normal function of several pathways, such as the HPG axis and genes involved in steroidogenesis, leading to changes in hormone levels associated with male reproduction, particularly testosterone.

### Female studies

Within the ovary, FSH and LH are critical regulators in the growth of preovulatory follicles and their transformation into corpora lutea. FSH binds to the follicle-stimulating hormone receptor (FSHR) on granulosa cells, while LH targets the luteinizing hormone receptor (LHR) on theca cells. Moreover, E2 and P4 are vital for the growth and differentiation of female reproductive organs and the maintenance of fertility [183]. E2 enhances steroidogenesis, promotes the proliferation of ovarian granulosa cells, and supports follicular development by influencing the expression of genes that regulate the cell cycle.

Exposure to F has been shown to reduce serum levels of LH, FSH, GnRH, T, and E2 in a dose-dependent manner, primarily due to decreased GnRH production [23, 32, 184] (Table 4) (Fig. 6). For instance, a study by Chen et al. [87] demonstrated that E2 release from KGN cells decreased at low NaF concentrations but increased at higher doses, indicating a complex relationship between NaF exposure and hormone secretion [87]. Further, Zhou et al. [185] found that female rats exposed to NaF showed decreased serum levels of E2 and P4, along with reduced expression of FSHR, while estrogen receptor alpha (ERα), progesterone receptor (PgR), and LHR expressions increased [185]. Research indicates that ERα is essential for fertility, while estrogen receptor beta (ERβ) does not play a significant role [186]. NaF appears to interfere with E2 binding to ERα, affecting receptor protein expression and reducing reproductive hormone levels, which may contribute to fertility impairments. Additionally, Jhala et al. [187] indicated that NaF exposure can disrupt ovarian steroidogenesis by inhibiting enzymes involved in steroid hormone conversion, particularly 3/17β-hydroxysteroid dehydrogenases (3/17β-HSDs) [187]. Li et al. [179] noted that NaF treatment in zebrafish led to dose-dependent changes in the expression of genes related to steroidogenesis, with increased PgR and Cyp19a1a but decreased 3β-HSD expression [179].

Therefore, these findings suggest that the impact of NaF on E2 production is influenced by concentration and compensatory mechanisms in steroidogenic pathways.

## Fluoride exposure and antioxidant therapy

Fluoride exposure occurs primarily through the consumption of fluoridated water, toothpaste, and various dental products, as well as its natural presence in many foods, raising concerns about its potential effects on human health. Furthermore, ROS have been implicated in mediating fluoride-induced toxicity within the reproductive system, leading to increased interest in pharmacological approaches to target oxidative stress. Among the numerous strategies available, one of the most prevalent involves the use of antioxidant compounds, including various plant-derived substances and vitamins, to mitigate oxidative stress (Fig. 4) [189, 190].

In this regard, *melatonin* administration (10 mg/kg) has been shown to counteract NaF-induced testicular injury in rats by enhancing antioxidant parameters, including GPx, GSH, SOD, GST, and CAT [191]. Also, *melatonin* treatment at the same dose for 1 month improved ovarian histopathology and increased body and ovarian weights in NaF-treated mice (10 mg/kg), elevating SOD, CAT, and GSH levels while reducing MDA levels (Fig. 4) [192]. Kumar et al. [65] demonstrated that *melatonin* downregulated NF-kB/COX-2 and upregulated SIRT-1/FOXO-1, Nrf2/HO-I, and connexin-43, mitigating NaF-induced testicular damage and improving sperm count and viability in golden hamsters [65].

In male LL-17A knockout C57BL/6 J mice, NaF treatment (24 mg/kg, for 3 months) decreased Nrf2 expression and increased ferroptosis, while riboflavin at 5 mg/kg remarkably reduced elevated ROS levels [193]. Parlak et al. [194] found that *royal jelly* protected against NaF-induced testicular damage via Nrf2 signaling activation [194].

Feng et al. [51] highlighted a strong correlation between oxidative damage and DNA damage in testicular tissues of rats treated with NaF (25 mg/kg, for 7 weeks), which decreased sperm count and quality while damaging serum testosterone levels, while *N-acetylcysteine* (NAC, 150 mg/kg) was reported to reduce 8-OHdG levels, alleviating these harmful effects [51]. Additionally, Hu et al. [73] noted that *NAC* at the same dose for seven weeks reduced NaF-induced testicular apoptosis by increasing nuclear Nrf2 expression and targeting IRE1α/JNK signaling in rats [73].

*Vitamin C*, *calcium*, and *vitamin E*—administered alone or in combination—effectively mitigated NaF's adverse effects on the female reproductive system by enhancing GSH-Px, SOD, and CAT activity while modulating apoptosis and inflammatory-related gene expression (Bcl-2, Bax, p53, TNF-α, and NF-κB) [35, 128, 195–197]. For example, *vitamin C* (100 mg/L) improved FSH, LH, and E2 levels in the Asian stinging catfish, Heteropneustes fossilis, and prevented ovarian damage from NaF exposure [198]. *Vitamin C* (50 µM) provided partial protection by elevating antioxidant enzymes (SOD, CAT, GPx, GST, and γ-GT) in TM4 Sertoli cells exposed to NaF (4 or 20 ppm for 24 or 48 h) [199]. Furthermore, a combination of *vitamin E* (20 mg/100 g body weight) and T (40 μg/100 g body) enhanced testicular weight and sperm count in NaF-exposed rats (20 mg/kg, for 1 month) [200]. Tian et al. [201] found that *vitamin E* may prevent spermatogenic cell apoptosis induced by coal-burning fluorosis through oxidative stress-mediated ERK and JNK signaling pathways [201]. Pal et al. [202] demonstrated that *vitamins C* and *E* at 200 and 400 mg/kg, respectively, reduced DNA damage in testicular and spermatozoa cells of rats exposed to NaF (15 mg/kg) [202]. Also, *vitamin B12* (0.63 µg/kg) countered degenerative testicular changes, improving sperm count, motility, viability, and semen volume while alleviating low T levels and elevated MDA and NO in NaF-treated rats (100 mg/L) [78].

Dibyendu et al. [203] reported that *folic acid* supplementation (36 μg/kg, for 3 weeks) improved sperm motility and viability while increasing serum LH and T levels, enhancing antioxidant parameters (SOD and CAT), and reducing inflammatory markers TNF-α and IL-6, alongside the alternation in the expression of 3β-HSD and 17β-HSD genes in rats subjected to NaF (100 mg/L) [203]. Lin et al. [54] found that *folic acid* (100 mg/L) improved oocyte ovulation rates in NaF-treated C57BL/6 mice (60 mg/L, for 20 days) through Sirt1/Sod2-dependent mechanisms [54].

Liu et al. [90] reported *Glycine’s* protective role against NaF-induced impairments in porcine Sertoli cells [90]. Treatment with *Nigella sativa L. oil* (NSO, 300 mg/kg, administered 2 weeks prior to NaF treatment [20 mg/kg], continued for 6 weeks) could improve serum E2, P4, FSH, and LH levels, preventing ovarian tissue injury in rats [204]. Qi et al. [205] identified *Honokiol's* protective effects on oocyte developmental potential and mitochondrial function impaired by NaF, through modulating SOD2/SIRT3 signaling pathways [205].

Deiab et al. showed that *Hesperidin/Chitosan Nanogel* (20 mg/kg) suppressed endoplasmic reticulum stress (PERK, ATF6, and IRE-α) and apoptosis (Bax, caspase-3, caspase-9, and P38MAPK), and reduced testicular damage in NaF-treated mice (10 mg/kg, for 1 month), while normalizing LH, FSH, and T levels [206]. Administration of *Epigallocatechin Gallate* (EGCG, 40 mg/kg for 1 month) could protect rats treated with NaF (25 mg/kg) by enhancing antioxidant levels (SOD, CAT, GPx, GST) and decreasing the expression of apoptosis and inflammation-related genes such as Bad, Cyt-c, Caspase-3, IL-1β, TNF-α, and COX-2 [64]. Wang et al. [207] demonstrated that *aluminum* (Al, 0.1 mg Al^3+^/kg, for 3 months) alleviated reproductive toxicity in NaF-male animals, characterized by elevated MDA and H_2_O_2_ levels, decreased calcium, iron, and magnesium, and increased expression of c-Fos, a redox-sensitive transcription factor involved in cell proliferation, cell death, and stress response in the testes [207]. Both *banaba leaf extract* and *ginseng extract* (150 mg/kg, over periods of 15 and 30 days) improved sperm parameters in NaF-exposed Swiss mice (600 ppm, for 1 month) [118]. *Polyphenol-rich nano Moringa oleifera* (250 mg/kg) balanced oxidants and antioxidants, restored sperm motility/viability, reduced morphological malformations, enhanced T and DHEA levels, upregulated the StAR gene expression, and improved histological features in NaF-exposed rats (10 mg/kg) [208]. Zhao et al. found that *sodium selenite* (Na_2_SeO_3_, 0.5–2.0 mg/L, for 10 weeks) elevated GPX-4, SePP, pregnenolone, androstenedione, and T levels, along with the mRNA and protein expression of steroidogenesis-related genes namely StAR, 3β-HSD, and 17β-HSD in NaF-treated rats (100 mg/L) in a dose-dependent manner [70].

Therefore, the compounds discussed have the capacity to enhance antioxidant defenses, restore hormonal balance, and mitigate oxidative stress, indicating their potential role in protecting reproductive health against the adverse effects of fluoride exposure.

## Conclusion and future prospects

Fluoride exposure is linked to reproductive abnormalities in both males and females. In males, it can cause testicular injury through oxidative stress, apoptosis, and inflammation, leading to impaired spermatogenesis and reduced sperm quality. In females, fluoride disrupts hormonal balance and mitochondrial function, resulting in ovarian injury, disrupted follicular development, and compromised oocyte quality. The effects of fluoride depend on dose, duration, and individual susceptibility. Future efforts should focus on understanding these mechanisms, enhancing regulations, raising public awareness, and exploring alternatives to fluoride that maintain benefits without reproductive risks.

## Data Availability

No data was used for the research described in the article.

## Abbreviations

F: Fluoride
NaF: Sodium fluoride
iNOS: Nitric oxide synthase
NO: Nitric oxide
SOD: Superoxide dismutase
CAT: Catalase
GSH: Reduced glutathione
GPx: Glutathione peroxidase
GR: GSH reductase
GST: GSH S-transferase
ROS: Reactive oxygen species
OS: Oxidative stress
MDA: Malondialdehyde
SePP: Selenoprotein-P
LPO: Lipid peroxidation
8-OHdG: 8-Hydroxy-2′ -Deoxyguanosine
HO-1: Hemeoxygenase-1
Nrf2: NF-E2-related factor 2
NF-κB: Nuclear factor-κB
COX-2: Cylooxygenase-2
JNK: C-Jun N-terminal kinase
FOXO-1: Forkhead box transcription factor O-1
TNF-α: Tumor necrosis factor-α
IL-6: Interleukin-6
3β-HSD: 3β-Hydroxysteroid dehydrogenase
17β-HSD: 17β-Hydroxysteroid dehydrogenase
P450c17: Cytochrome p450 17α hydroxylase
P450scc: Cytochrome P450 cholesterol side chain lyase
StAR: Steroidogenic acute regulatory protein
AR: Androgen receptor
E2: Estradiol
T: Testosterone
LH: Luteinizing hormone
FSH: Follicle stimulating hormone
P4: Progesterone
LHR: Luteinizing hormone receptor
ERα: Estrogen receptor alpha protein
PgR: Progesterone receptor
FSHR: Follicle-stimulating hormone receptor
BTB: Blood-testis barrier
HPG: Hypothalamic-pituitary-gonadal
GnRH: Gonadotropin-releasing hormone
SHBG: Sex hormone-binding globulin
SIRT-1: Silent information regulator transcript-1
LDH: Lactate dehydrogenase
ACP: Acid phosphatase
ALP: Alkaline phosphatase
GLUT-1: Testicular glucose transporter
GSH-R: Glutathione reductase
GRP78: Glucose-regulated protein 78
